# Comparison of the Convolution algorithm with TMR10 for Leksell Gamma knife and dosimetric verification with radiochromic gel dosimeter

**DOI:** 10.1002/acm2.12238

**Published:** 2017-12-10

**Authors:** Petra Osmancikova, Josef Novotny, Jaroslav Solc, Jan Pipek

**Affiliations:** ^1^ Department of Dosimetry and Application of Ionising Radiation Faculty of Nuclear Sciences and Physical Engineering CTU in Prague Prague Czech Republic; ^2^ Na Homolce Hospital Prague Czech Republic; ^3^ Department of Radiation Oncology University Hospital Motol Prague Czech Republic; ^4^ Czech Metrology Institute Brno Czech Republic

**Keywords:** gel dosimetry, stereotactic radiosurgery, verification

## Abstract

The Convolution algorithm, implemented in Leksell GammaPlan^®^ ver. Here, 10, is the first algorithm for Leksell Gamma Knife that takes heterogeneities into account and models dose build‐up effects close to tissue boundaries. The aim of this study was preliminary comparison of the Convolution and TMR10 algorithms for real clinical cases and dosimetric verification of the algorithms, using measurements in a phantom. A total of 25 patients involved in comparison of the Convolution and TMR10 algorithms were divided into three groups: patients with benign tumors close to heterogeneities, patients with functional disorders, and patients with tumors located far from heterogeneities. Differences were observed especially in the group of patients with tumors close to heterogeneities, where the difference in maximal dose to critical structures for the Convolution algorithm was up to 15% compared to the TMR10 algorithm. Dosimetric verification of the algorithm was performed, using a radiochromic gel dosimeter based on Turnbull blue dye in a special heterogeneous phantom. Relative dose distributions measured with the radiochromic gel dosimeter agreed very well with both the TMR10 and Convolution calculations. We observed small discrepancies in the direction in which the largest inhomogeneity was positioned. Verification results indicated that the Convolution algorithm provides a different dose distribution, especially in regions close to heterogeneities and particularly for lower isodose volumes. However, the results obtained with gamma analyses in the gel dosimetry experiment did not verify the assumption that the Convolution algorithm provides more accurate dose calculation.

## 1 INTRODUCTION

The Leksell Gamma Knife^®^ Perfexion™ stereotactic radiosurgery system operating with submillimeter accuracy offers two‐dose calculation algorithms for Leksell GammaPlan^®^ (LGP): TMR10 and a Convolution algorithm. Until the Convolution algorithm was introduced in LGP, the radiosurgery treatment planning system ignored heterogeneity corrections and dose calculations were based solely on attenuation in water. Brain tissue is relatively homogeneous but beams pass through air‐filled cavities and bones, and substantial differences can exist in the dose distribution when homogeneity is not assumed.^1^ Moskvin et al. (2004) reported that the dose delivered to the target area is underestimated by up to 7% by the algorithm assuming homogeneous media geometry.^2^ In the Convolution algorithm, an added software module enables accurate dose calculation for treatment of heterogeneous tissue. The algorithm takes into account build‐up effects as well as heterogeneity effects.^3^

The aim of this study was the preliminary comparison of the Convolution algorithm with TMR10 on real clinical cases and dosimetric verification. Verification of a new dose calculation algorithm should precede its implementation in routine clinical use.

In stereotactic radiosurgery, the selection of a suitable detector is not a trivial task. Gel dosimeters, as truly 3D dosimeters, are a promising tool for three‐dimensional dose measurements in high‐dose‐rate radiosurgery. Polymer gel dosimetry with PAGAT (Polyacrylamide gelatine gel fabricated at atmospheric conditions) and magnetic resonance imaging have a long history of use for this application.^4^ However, temperature stabilization, toxicity, and access to an MR scanner for evaluating the PAGAT gel response makes radiochromic gel dosimeters more user‐friendly for clinical applications.^5^ A radiochromic gel dosimeter based on Turnbull blue dye formed by irradiation (TB gel) is an integral chemical dosimeter introduced by Solc et al.^6^ The response can be evaluated, using both cone‐ and laser‐beam optical CT. Basic properties of the TB gel dosimeter are summarized by Solc et al.^7^ and Vavru et al.^8^ It offers several advantages such as inhibited diffusion, linear response from 0 Gy up to at least 400 Gy, easy preparation, and nontoxic composition. The main disadvantages of TB gel dosimeters are lower sensitivity that requires a longer irradiation time, and gel aging caused by the spontaneous interactions of ferric ions with organic compounds of the gel. Gel response is stable if the gel is refrigerated in the dark, and evaluation of the irradiated TB gel should be performed within a few days following irradiation.

## 2 MATERIALS AND METHODS

### 2.A Algorithm comparison for clinical cases

**Figure 1:**
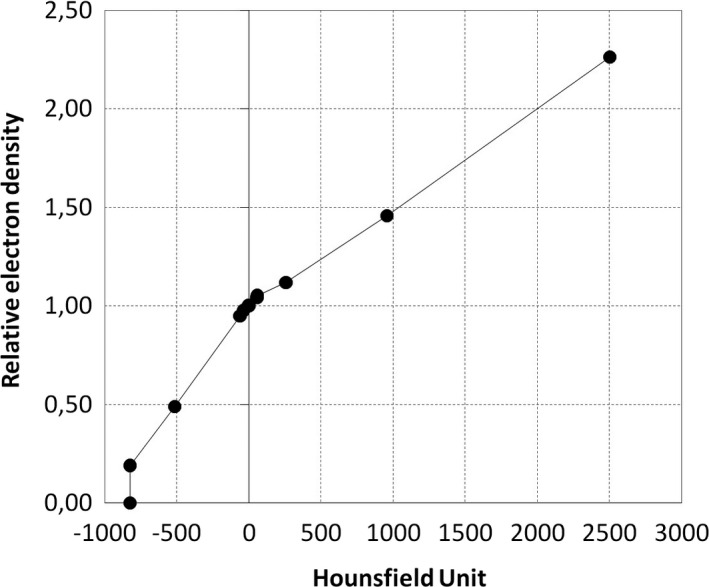
Calibration curve for CT scanner.

As required for implementation of the Convolution algorithm, a calibration curve for the CT scanner (Somatom Definition Flash; Siemens Healthcare GmbH, Erlangen, Germany) was measured, using a CIRS Electron Density Phantom Model 062M (Head Insert) (Norfolk, VA, USA) with eight different tissue equivalent inserts, using a head protocol (120 kVp, 1 mm slice thickness). For clinical implementation of the Convolution algorithm, the calibration curve between Hounsfield units and relative electron density had to be adjusted to avoid problems with streaking artifacts at the edge of the CT scan. Relative electron densities lower than ~0.2 were considered to be air, and since a high density insert was not available for the CIRS phantom, the calibration curve was extrapolated to include the high density fixation screws (Fig. 1).

A total of 25 patients were involved in the clinical testing of the Convolution algorithm. Patients were divided into three groups: Group 1 (12 patients) with benign tumors close to heterogeneities (vestibular schwannomas and pituitary adenomas); Group 2 (7 patients) with functional disorders (trigeminal neuralgia); Group‐3 (6 patients) underwent a full‐head CT scan and contained mostly patients with centrally located brain metastases, uveal melanomas, or meningiomas far from heterogeneities.

A treatment plan was created for each patient, using the TMR10 algorithm and then recalculated with the Convolution algorithm keeping all other planning parameters fixed. Treatment plan parameters are summarized in Table 1. Various parameters were used for the comparison: isodose volumes (prescription isodose, 30% and 20% isodoses), tumor coverage, Paddick conformity index,^9^ gradient indexes, treatment time, and doses to critical structures.

**Table 1:** Parameters of treatment plans for 25 patients was involved in clinical testing of the Convolution algorithm

Diagnoses	Number of patients	Mean prescription dose (Gy)	Mean prescription isodose (%)	Mean planning isodose volume (mm^3^)
Pituitary adenoma	6	18.7 (range 14–35)	50.1 (range 50–54)	2940.7
Vestibular schwannoma	6	12.6 (range 12–13)	50	1321.7
Trigeminal neuralgia	6	64	80	31.0
Meningioma	2	21 (range 20–22)	44	23550.0
Metastases	4	21 (range 20–22)	64.5 (range 48–80)	3290.0
Uveal melanoma	1	35		301.7

### 2.B Gel dosimetry verification

A TB gel was prepared, using the procedure suggested by Solc et al.^7^ It is composed of a gel matrix – 0.25% (w/w) phytagel, 0.5 mM potassium ferricyanide, and 0.5 mM ferric chloride compound dissolved in 1 mM sulphur acid medium. The prepared gel was poured into two glass cylindrical flasks (0.2 l, with a diameter of 6 cm), with one gel sample used for irradiation and one sample for background. Gel dosimeters were stored in a refrigerator at 5 °C. Solidification of the TB gel took roughly 48 h.

**Figure 2:**
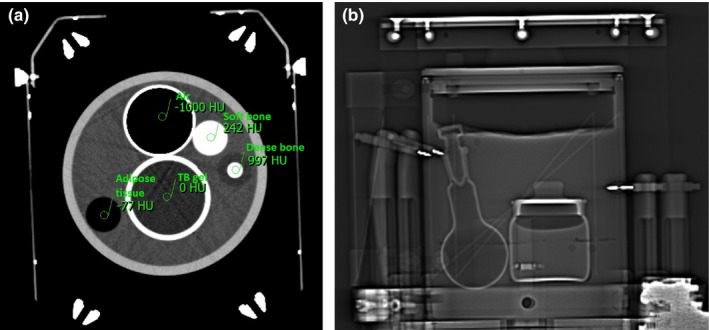
Transverse CT scan (a) and side projection (b) of heterogeneous phantom with TB gel dosimeter.

Verification of the new algorithm required design of a special heterogeneous phantom. One of the gel dosimeters described above was placed in a large cylindrical volume (height 15.13 cm, outer diameter 14.95 cm) and surrounded with several inhomogeneities; a glass flask with air, and small cylinders representing dense, and soft bone and adipose tissue. The rest of the phantom volume was filled with gelatin from porcine skin. The Leksell stereotactic head frame was attached to the phantom, using four aluminum fixation screws to reproduce patient immobilizations as closely as possible. The CT indicator was attached to the frame to provide the fiducial markers necessary for coordinate system definition in the LGP system and the phantom was scanned. A transverse CT image and phantom side projection are shown in Fig. 2.

The phantom shape (skull definition) and electron densities were defined, using the CT data. A treatment plan was generated, using the TMR10 algorithm. The phantom was irradiated with a single 8‐mm shot, positioned at the center of the TB gel dosimeter to a prescription dose of 50 Gy to the 50% isodose, requiring a treatment time of 60.6 min for a dose rate of 1.873 Gy/min. Irradiation took place in a dark treatment room. A second, nonirradiated gel sample was stored separately in a dark at room during the experiment at the same temperature. An identical plan was recalculated with the Convolution algorithm and both plans were exported in DICOM format.

The TB gel dosimeter was removed from the phantom and scanned, using a homemade optical CT scanner 2 h after irradiation.^10^ The nonirradiated gel sample was also scanned to obtain a background reading. The optical CT scanner, described in more detail by Solc et al.,^11^ consisted of a 16‐bit astronomy CCD camera (G2‐0402 type, Moravian Instruments, Czech Republic), stepper motor (65535 micro‐steps per 360 turn), and a light source (a red diode array emitting light at a peek wavelength of 660 nm). Data analysis was performed, using software developed in‐house in the Matlab^®^ environment (The MathWorks, Inc., Natick, MA, USA), as suggested by Solc et al.^7^ The reconstructed image had a resolution of 4.2 px/mm.

The treatment planning system (TPS) dose distributions exported from LGP were imported into Matlab^®^. The background map was determined from the nonirradiated gel sample and subtracted from the irradiated dosimeter to reduce scatter artifacts arising at the gel–glass interface. Dose maps were normalized to the value corresponding to the center of the shot.

## 3 RESULTS

### 3.A Algorithm comparison for clinical cases

**Figure 3:**
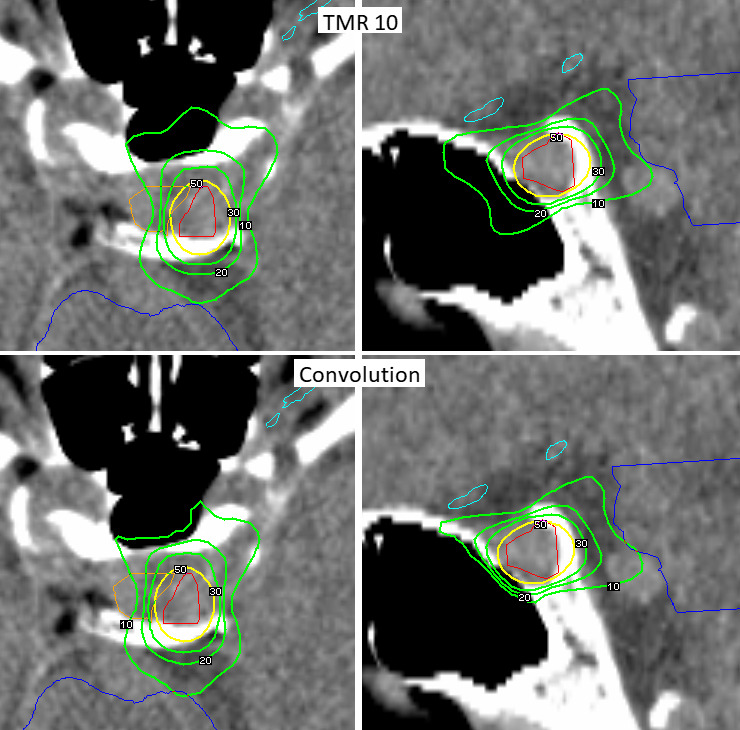
Dose distribution comparison for pituitary adenoma radiosurgery treatment plan, TM10 and Convolution algorithms. Treatment isodose (yellow), 30%, 20% and 10% isodoses (green), target volume (red), brain stem (dark blue), optics (light blue), pituitary gland (orange).

Differences between the two algorithms were observed particularly in Group 1, with tumors close to heterogeneities. Differences between the two algorithms are shown in Fig. 3 on dose distribution for pituitary adenoma treatment. We observed that the Convolution algorithm reduced the prescription isodose volume by 2.4% (range −4.8% to −1.2%), and in the case of 30% isodose and 20% isodose, by 4.3% (range −8.3% to −1.8%), and 5.3% (range −10.5% to −1.6%), respectively. Tumor coverage decreased by 0.7% (range −2.1% to 0%), Gradient index decreased by 2.9% (range −12.6% to 0.4%) and the Paddick Conformity index increased on 2.9% (range −0.9% to 7.1%). Treatment time increased by 3.2%. Deviation in maximal doses to 1 mm^3^ of critical structures observed in the Convolution recalculated plans decreased by up to 10.1% for the optic nerve, increased up to 3.9% for the brain stem in case of pituitary adenomas, and decreased up to 15.6% for cochlea in case of vestibular schwannomas.

In Group 2, prescription isodose volume increased by 3.1% (range 2.2% to 3.7%), while 30% isodose and 20% isodose volumes decreased by 2.7% (range −4.9% to −1.8%) and 3.4% (range −5.6% to −2.4%), respectively. Treatment time increased by 2.7%. For Group 3, the average differences in isodose volumes, gradient, and conformity indices were less than 2.0%. Treatment times in Group 3 increased by 3.8%.

### 3.B Gel dosimetry verification

**Figure 4:**
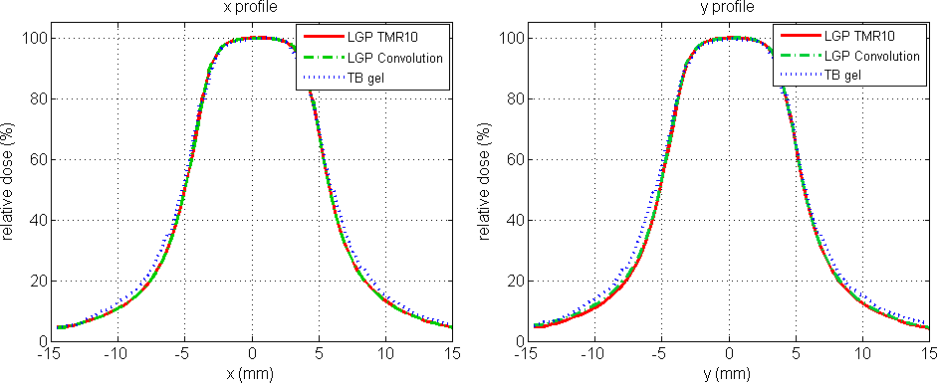
Comparison of relative central x and y profiles for the TB gel dosimeter, TMR10 and Convolution algorithms.

**Figure 5:**
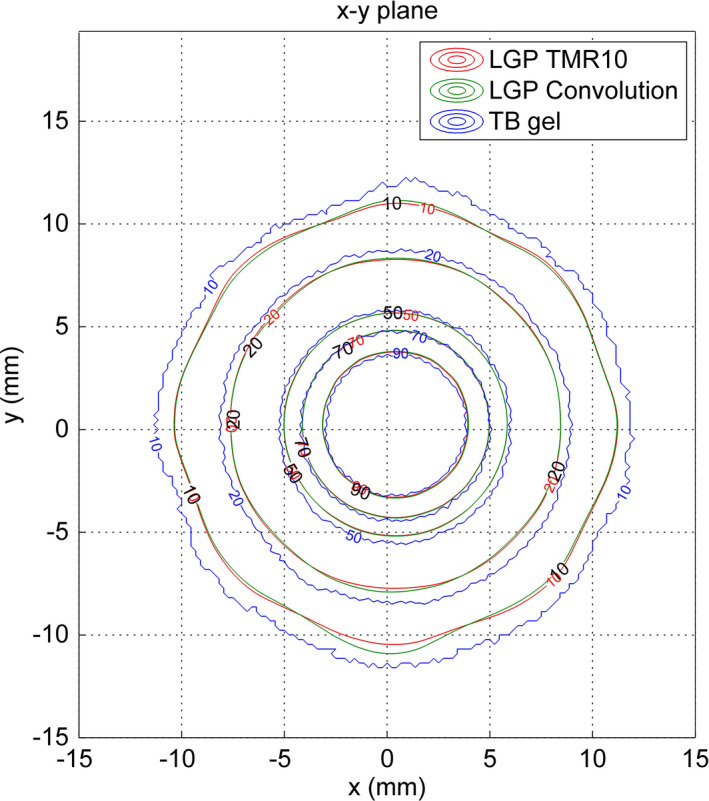
Comparison of 2D relative dose distributions. The red lines are Leksell GammaPlan TMR10 calculations, the green lines are Convolution calculations and the blue lines are TB gel measurements. Displayed isodoses: (10%, 20%, 50%, 70% and 90%).

**Figure 6:**
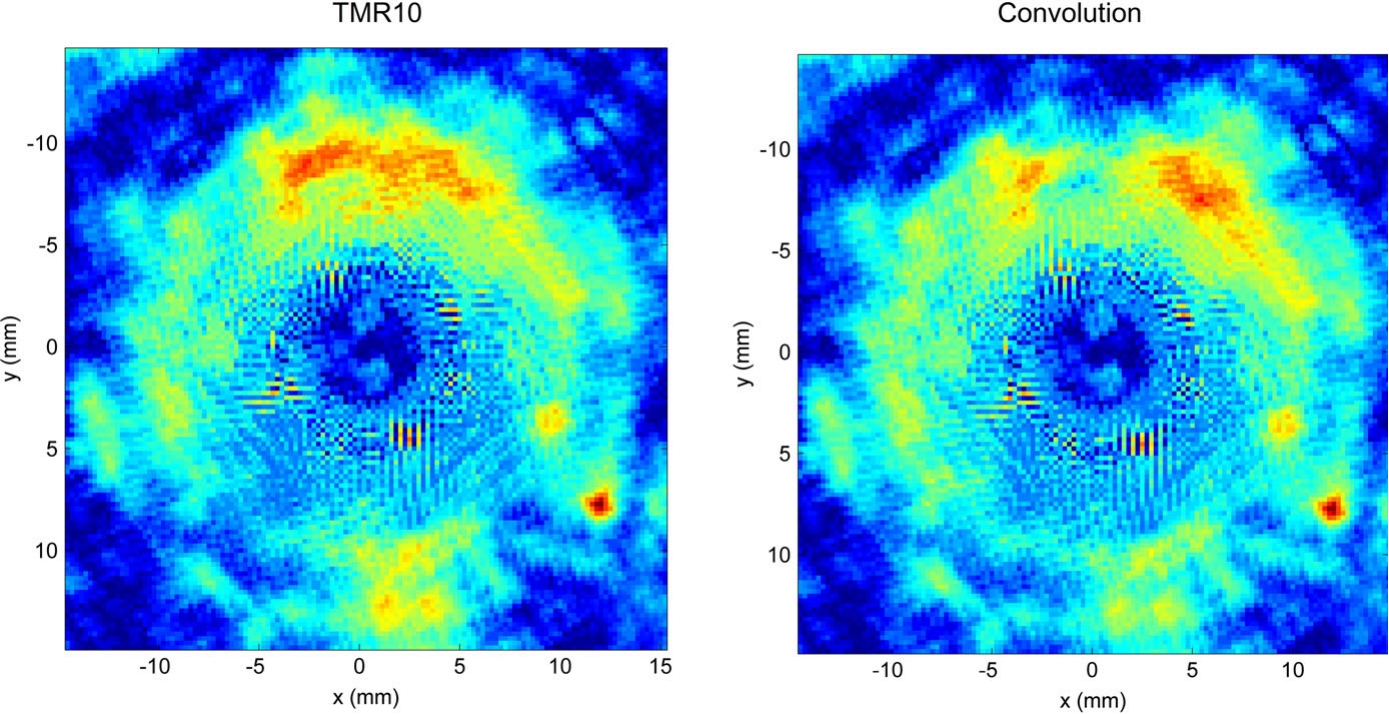
Two‐dimensional gamma analyses (1 mm, 3%) in central slice of the gel for TMR10 (left) and Convolution (right).

**Figure 7:**
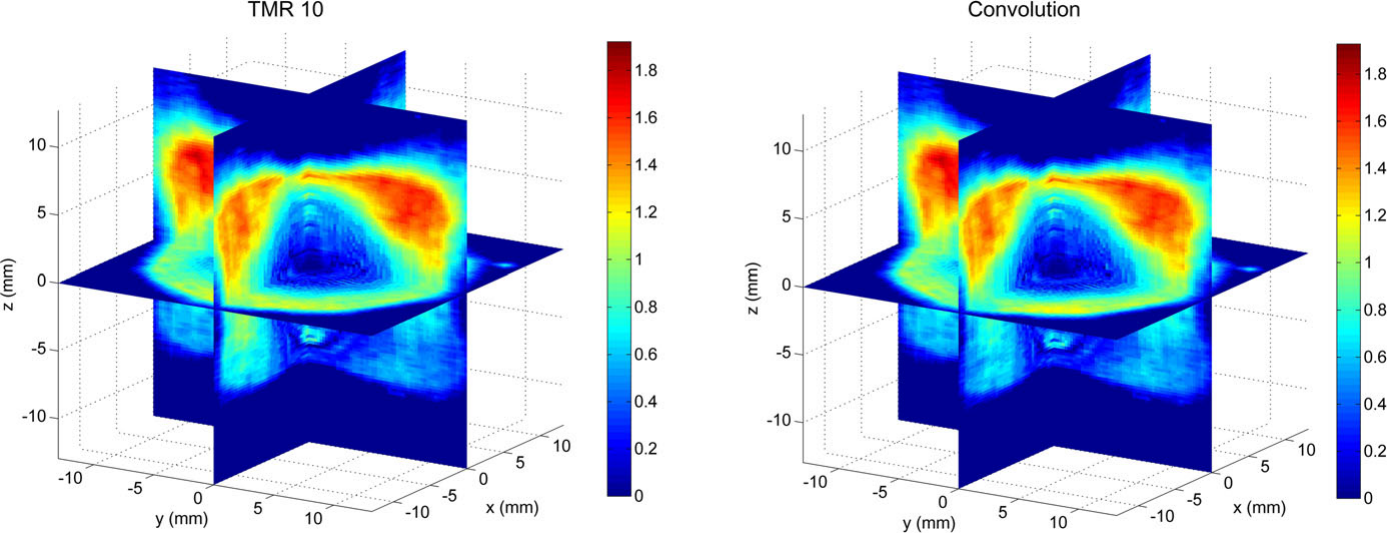
Three‐dimensional gamma analyses in a 30 × 30×30 mm^3^ gel volume (1 mm, 3%, dose threshold 10%) for TMR10 (left) and Convolution (right).

Comparisons between gel measurements and TPS calculations are presented in the form of 1D *x* and *y* profiles (Fig. 4), 2D isodoses (Fig. 5), 2D gamma maps with 3% dose difference, and 1 mm distance to agreement criteria calculated for the central slice (Fig. 6) and three‐dimensional gamma analyses, using 3% dose difference and 1 mm distance to agreement and 10% dose threshold criteria (Fig. 7). The measured relative dose distributions agreed very well with the both TMR10 and Convolution calculations. We observed small discrepancies in the direction of the *y* axes, where the largest inhomogeneity (air filled flask) was positioned, and in the *z* direction (the remainder of the upper part of the gel dosimeter was filled with air). However, the results obtained by gamma analyses show minimal differences between both algorithms. Two‐dimensional gamma analyses (1 mm, 3%) in central slice for TMR10 calculations showed gamma area >1 was 1.21%, gamma maximum 1.0144, gamma average 0.3174; for Convolution calculations gamma area >1 was 1.17%, gamma maximum 1.019 and gamma average 0.3210. Results of three‐dimensional gamma analyses (1 mm, 3%, dose threshold 10%) in a 30 × 30 × 30 mm^3^ volume of gel for TMR10 calculations showed gamma area >1 was 12.918%, gamma maximum 1.9227, gamma average 0.3893, and for Convolution gamma area >1 was 12.990%, gamma maximum 1.9294 and gamma average 0.3963.

## 4 DISCUSSION

Clinically relevant differences in dose distributions were observed for patients with a target located close to heterogeneities (e.g., pituitary adenomas and vestibular schwannomas). Differences were mostly visible for lower isodose lines, for example, 20% isodose volume changed by 5.5%, and for maximal dose to critical structures, for example, dose reduction to the cochlea up to 15.6%. For patients with targets located in homogeneous areas, minimal differences were observed. These results are in agreement with gel dosimetry verification where larger discrepancies between the two algorithms were observed only in the low dose region. Attenuation of a beam passing through air is underestimated and the dose is increased away from the interface. This can result in clinically relevant differences in dose distributions for patients with a target located close to heterogeneities. Treatment planning in stereotactic radiosurgery operating with submillimeter accuracy works on the assumption that the dose calculation is accurate. Dose to a critical structure, located at the level of lower isodose, can differ significantly for the Convolution calculation. However, results obtained with gamma analyses in the gel dosimetry experiment do not verify the assumption that the Convolution algorithm provides more accurate dose calculation. This is in agreement with results presented by Novotny et al.^12^ They measured mean dose in an anthropomorphic head phantom for a clinical test plan calculated by both Leksell GammaPlan TMR10 and Convolution algorithms with PTW 31010 ion chambers. They observed deviations within the target volume of −1.1% and 2.5% for TMR10 and Convolution algorithms, respectively.

The main limitation of the gel study is the impossibility of placing inhomogeneities directly into the gel dosimeter, as the gel should be enclosed in a container suitable for optical CT scanning. The irradiated volume was positioned in the center of the gel dosimeter due to scatter artifacts from glass wall of flask. Using flasks with walls from nonscattering material would be more appropriate.

Measurement of absolute dose for the TB gel is not well established yet, mainly because of issues associated with gel aging. Temperature and light history, as well as time intervals between preparation, irradiation and evaluation has to be identical for both calibration samples and the evaluated volume.

## 5 CONCLUSION

Preliminary experience indicates that the Convolution algorithm, which incorporates tissue inhomogeneities, provides a different dose distribution, especially in areas close to heterogeneities, particularly for lower isodose volumes. This affects some treatment plan parameters, especially doses to critical structures, where the difference in maximal dose to critical structures in the Convolution algorithm was up to 15% compared to the TMR10 algorithm. Relative dose distributions measured with the TB gel agreed very well with both TMR10 and Convolution calculations. Small discrepancies were observed in the direction in which the largest inhomogeneity was positioned. The assumption that the Convolution algorithm provides more accurate dose calculation was not verified and verification of Convolution algorithm accuracy remains a future task for Monte Carlo simulation.

## CONFLICT OF INTEREST

Josef Novotny Jr. is employed as consultants of Elekta Instruments AB, Stockholm.

## References

[1] Solberg TD , DeMarco JJ , Holly FE , Smathers JB , DeSalles AA . Monte Carlo treatment planning for stereotactic radiosurgery. Radiother Oncol J Eur Soc Ther Radiol Oncol. 1998;49:73–84. 10.1016/s0167-8140(98)00065-6 9886701

[2] Moskvin V , Timmerman R , DesRosiers C , et al. Monte Carlo simulation of the Leksell gamma knife^®^: II. Effects of heterogeneous versus homogeneous media for stereotactic radiosurgery. Phys Med Biol. 2004;49:4879. 15584525 10.1088/0031-9155/49/21/003

[3] Elekta . The Convolution Algorithm in Leksell GammaPlan 10 [white paper]. Stockoholm, Sweden: Elekta.

[4] Baldock C , et al. Polymer gel dosimetry. Phys Med Biol. 2010;55:R1–R63. 20150687 10.1088/0031-9155/55/5/R01 PMC3031873

[5] Vandecasteele J , Deene YD . Evaluation of radiochromic gel dosimetry and polymer gel dosimetry in a clinical dose verification. Phys Med Biol. 2013;58:6241. 23965800 10.1088/0031-9155/58/18/6241

[6] Šolc J. Vývoj nových typů radiochromních gelových dozimetrů a vyhodnocení jejich odezvy metodou optické výpočetní tomografie, disertační práce, ČVUT v Praze, Praha; 2007.

[7] Šolc J , Spěváček V . New radiochromic gel for 3D dosimetry based on Turnbull blue: basic properties. Phys Med Biol. 2009;54:5095–5107. 19652291 10.1088/0031-9155/54/17/002

[8] Pilařová (Vávrů) K , Kozubíková P , Šolc J , Spěváček V . Characteristics of polyacrylamide gel with THPC and Turnbull Blue gel dosimeters evaluated using optical tomography. Radiat Phys Chem. 2014;104:283–286.

[9] Paddick I . A simple scoring ratio to index the conformity of radiosurgical treatment plans. J Neurosurg. 2000;93:219–222. 11143252 10.3171/jns.2000.93.supplement

[10] Šolc J , Sochor V . Feasibility of radiochromic gels for 3D dosimetry of brachytherapy sources. Metrologia. 2012;49:S231.

[11] Šolc J , Sochor V , Kačur M , Šmoldasová J . 3D dose distribution measurements in brachytherapy using radiochromic gel dosimeters. Nucl Instrum Methods Phys Res A. 2010;619:163–166.

[12] Novotný J Jr. , Koniarová I , Horáková I. Dosimetry audit and comparison of two calculation algorithms for Leksell Gamma knife. In: 13th International Stereotactic Radiosurgery Society Congress, Abstract Book, Montreux, Switzerland; 2017.

